# Ovariotomy with Extirpation of One Kidney

**Published:** 1884-12

**Authors:** 


### Ovariotomy with Extirpation of one Kidney.

The *Journal of the American Medical Association* has information that Prof. Donald Maclean, of Detroit, recently extirpated the left kidney, both ovaries, and a large piece of the omentum, from a married woman, aged 27 years, who proved to be in an early stage of pregnancy. The kidney and and both the ovaries were cystic, the former being very large.

The operation was done October 26, 1884, at Ann Arbor, Mich., in the presence of the medical class and a number of physicians; one week afterwards the patient was doing well, and the case promised well for success.

P. S.—Two weeks more have elapsed since the operation, and we learn, just as the Journal goes to press, that the case has continued to progress favorably, and is now considered convalescent. We hope to publish a full history of this remarkable case in a future number.

